# Risk of Postpartum Depression: The Considerable Role of Maternal Health Status and Lifestyle

**DOI:** 10.3390/healthcare11142074

**Published:** 2023-07-20

**Authors:** Haya S. Zedan, Baian A. Baattaiah, Shoug Alashmali, Arwa S. Almasaudi

**Affiliations:** 1 Department of Public Health, College of Health Sciences, Saudi Electronic University, Riyadh 13316, Saudi Arabia; 2 Department of Physical Therapy, Faculty of Medical Rehabilitation Sciences, King Abdulaziz University, Jeddah 21589, Saudi Arabia; 3 Department of Clinical Nutrition, Faculty of Applied Medical Sciences, King Abdulaziz University, Jeddah 21589, Saudi Arabia

**Keywords:** postpartum depression, women’s health, maternal health, mental health, lifestyle, Saudi Arabia

## Abstract

Women’s health issues are complex and require collaborative efforts to unravel some of these complexities. This study aims to identify the incidence risk of Postpartum Depression (PPD) in a national sample of women in Saudi Arabia and the relationship with several health status, lifestyle, and sociodemographic factors. A cross-sectional study with an online questionnaire format assessed the risk of postpartum depression using the Edinburgh Postnatal Depression Scale (EPDS) and included several questions on postpartum health status and lifestyle factors. Of the 550 women who responded to the survey 75% scored within range of risk for PPD (≥12). We found significant associations between family income, younger baby age, birth difficulty, having family support, level of physical activity, and the risk for PPD (*p* < 0.05). Urgent attention and resources should be directed towards screening and treatment for PPD in the healthcare system. The development of programs for awareness, education, and support of postpartum mothers in the Kingdom of Saudi Arabia is also required.

## 1. Introduction

The World Health Organization (WHO) prioritizes a healthy diet, that is, nutrition, weight management, physical activity, planned pregnancy, and physical, mental, and psychosocial health for women of reproductive age [1,2,3,4,5]. (Women’s health issues are complex, and collaborative efforts are required to unravel some of these complexities in order to support women through their reproductive experiences, to enhance health and well-being, and to ensure better maternal outcomes [6,7,8,9]. Postpartum depression (PPD) is a common issue among childbearing women, as up to one in seven women will experience PPD [8,10,11]. PPD may develop within the first few weeks after a child’s birth and can seriously impact women’s health and well-being if left unrecognized and untreated [6]. PPD may also have a significant impact on women’s ability to bond with their babies, breastfeed or care for them, or care for themselves and their families [6,8,11].

Common symptoms of PPD include lack of interest or enjoyment, loss of appetite or ability to sleep, anxiety, racing or scary thoughts, difficulty concentrating or in decision-making, feelings of guilt or worthlessness, self-blame, excessive irritability, anger or agitation, crying, sadness or misery, fear of not being a good enough mother or of being left alone with the baby, or thoughts of harming the baby or self-harm [6,10,11]. If these symptoms persist longer than two weeks after delivery, professional help may be required, and recovery is possible with proper treatment and support [6,9,10,11]. PPD can affect all women, whether they were involved in low- or high-risk pregnancies, are first-time mothers, or have more than one child [6,10,11].

There are several well-documented risk factors that may exacerbate the potential development of PPD, including a prior history of depression and anxiety (either in pregnancy or previously) [6,8,11,12], a family history of mental illness, low levels of spousal support or family support [7,11,12,13,14], isolation [7,15,16], infant health issues (including infants with special needs) or breastfeeding and maternal health challenges (including mode of birth or delivery, such as caesarean section or birth trauma) [12], unplanned pregnancy [12,13,14], difficulties with breastfeeding [12], chronic health issues, fatigue and poor sleep quality [3,14,17,18], significant life events (e.g., the death of a loved one, moving houses or job changes), and financial troubles [11,12,19,20].

Much remains undiscovered about the health and lifestyle-related factors that may contribute to the risk of PPD among mothers living in Saudi Arabia, especially during emergency events, such as the COVID-19 pandemic. Previous studies from Saudi Arabia have reported increasing prevalence rates of PPD [13,15,17,19,20]. Most recently, a prevalence rate of 75.5% was found, associated with the number of weeks since birth, lack of family support, and unplanned pregnancy [13]. Another study has also found a similar rate (75%) that was significantly associated with fatigue, sleep quality, and resilience [17]. Previously, PPD rates of 38.5% have been significantly associated with having an unsupportive spouse [19]. A study from 2017 found a prevalence rate of over 50% that was significantly associated with maternal age, level of education and employment, monthly income, medical issues, unplanned pregnancy, and previous mental health issues [20]. We propose that the prevalence rate of PPD may be higher than detected in the studies reviewed, as many women may face these issues alone and do not report their struggles to healthcare providers due to societal expectations and concerns around the stigma associated with mental health issues [6,8,9,16,21].

Several international studies have discussed lifestyle factors associated with better perinatal mental health outcomes that are considered major safeguards, such as maternal diet, physical activity, and behavioral strategies, e.g., smoking cessation, anxiety/stress reduction, and sleep quality improvement [5,12,22,23,24,25,26,27]. Research has also addressed the impact and importance of maternal age, level of education, and social support in mitigating the risk of poor maternal mental health and wellbeing [8,11,12,16,28].

A large study in Brazil found that women with pregestational obesity had over 20% higher odds of developing PPD than those with normal weight [29]. A systematic review on the role of maternal dietary patterns and the potential risks of PPD found an inverse association where the healthier a woman’s diet was in the postpartum period, the lower the risk of developing PPD symptoms [30]. A balanced maternal diet consisting of fruits, vegetables, fish, grains, legumes, and herbs could potentially reduce the incidence of PPD [30]. A recently published chapter on women’s nutrition and mental health turned attention to a healthy diet and nutritional supplementation as a means to alleviate or prevent symptoms of mental disorders during the perinatal period [31]. Therefore, the health status of mothers contributes to their risk of PPD, and further research exploring the potential health- and wellness-related factors is necessary.

According to the WHO, the rates of PPD are increasing exponentially and must be addressed more proactively by healthcare professionals through the use of integrative approaches within women’s health [2,3,6,9,32]. The COVID-19 pandemic significantly increased the risk of PPD in women [7,9,33,34]. If undetected and untreated, the consequences of PPD are far-reaching and pose an urgent public health concern that will have serious economic implications [7,33,35].

PPD is still not screened for regularly in Saudi Arabia. This study sought to identify the incidence and risk of PPD among a national sample of women based on several health status, lifestyle, and sociodemographic factors. We assessed any potential relationship between these factors and the risk of PPD development. The findings will guide recommendations to support women in achieving more positive postpartum experiences and to raise awareness and draw attention to the importance of maternal mental health and well-being.

## 2. Methods and Methodology

### 2.1. Study Design and Sample Size Calculation

We conducted a cross-sectional survey using an online questionnaire format. Responses were collected from a national sample of mothers living in Saudi Arabia from April to September 2021. *A priori* sample size calculation was conducted using OpenEpi.com. Based on the total Saudi female population in 2020 [36] and setting the confidence level at 95%, the margin of error at 5%, and the anticipated frequency at 50%, the estimated sample size was 385 respondents.

### 2.2. Study Respondents and Recruitment

The inclusion criteria were female adults (18–50 years old) within 1 year postpartum and residing in Saudi Arabia, regardless of nationality. Respondents who did not meet these criteria were excluded. The online survey was delivered using Survey Monkey (Palo Alto, CA, USA). A thorough description of the study detailing the study protocol and respondents’ rights was provided at the outset of the online survey. All respondents were given the option to provide digital informed consent to participate by clicking yes or no before answering the survey questions. All respondents were free to end their participation at any point during the survey. The questionnaire was disseminated via social media platforms (e.g., Twitter v.8.89, WhatsApp v. 2.21.8.12, and e-mail) and required approximately 10 min to complete.

### 2.3. Study Instrument

The online questionnaire consisted of several items on sociodemographic, health status, and lifestyle indicators. Data related to age, gender, marital status, nationality, region, educational level, employment status, body mass index (BMI), income level, postpartum months, type of birth experience, breastfeeding status, pregnancy and birth issues, family support, smoking status, physical activity, history of mental illness, history of chronic illness, and recent use of antibiotics were collected. In addition, the Edinburgh postnatal Depression Scale (EPDS) screening tool for identifying depression was included in the survey. The survey was developed and reviewed by a group of healthcare professionals and piloted on a small group from the public before full release, as part of a series of projects by our research team [37,38].

### 2.4. Edinburgh Postnatal Depression Scale (EPDS)

The EPDS is a 10-item self-reported scale that assesses common symptoms of depression scored on a four-point scale (0–3), with total scores ranging from 0 to 30 [39]. The EPDS is a validated screening tool for PPD that has been translated into over 18 languages, including Arabic [40,41]. The Arabic version of the EPDS has a Cronbach’s alpha of 0.84, indicating good internal consistency. A value ≥ 12 has been shown to indicate good sensitivity and specificity in Arabic-speaking cultures [40,41,42]. Therefore, we considered the risk of PPD to be present in scores of 12 or higher on the EPDS scale [39,42].

### 2.5. Statistical Analysis

The statistical analysis was performed using IBM SPSS (version 28.0.1.0). In the data-cleaning process, no missing data related to the study’s main variables were identified. Data were presented as the mean (M) and standard deviation (SD) for continuous variables and as frequencies and percentages for categorical variables. Frequency distribution was used to determine the percentages of those at risk of PPD across various sociodemographic and general health variables.

Chi-square and Fisher’s exact tests were conducted to evaluate the association between demographic variables and the risk of PPD. Fisher’s or chi-square tests were used in the case of 2 × 2 contingency tables, while Pearson’s likelihood ratio was used for larger tables. Simple and multivariate logistic regression was used to assess health status, lifestyle, and sociodemographic variables as predictors of risk of PPD. Several variables were recoded into binary categories to reduce the likelihood of multicollinearity. Those that were difficult to categorize into two were converted into dummy variables. Only variables that returned significant results as PPD predictors from the simple regression were further entered into multivariate logistic regression.

To conduct the logistic regression, several demographic variables were recoded into binary categories: age of infant (≤6 months/>6 months), age of mother (<30/≥30), family income (≤10,000/>10,000 Saudi Riyal (SAR), birth difficulty (<4/≥4) where ≥4 combined “difficult” and “very difficult” and where <4 combined “very easy”, “easy”, and “moderate” categories), as well as education (below university level/university degree level). Variables that were difficult to categorize into two were converted into separate standalone variables (dummy variables).

### 2.6. Ethics Approval and Consent to Participate

The study was conducted in accordance with the guidelines proposed in the Declaration of Helsinki and all relevant local and international ethical guidelines and regulations. The study was reviewed and approved by the Unit of Biomedical Ethics Research Committee, Faculty of Medicine, King Abdulaziz University, Jeddah, Saudi Arabia (Registration Number: HA-02-J-008, Reference No 84-21). The study protocol, procedures, and respondents’ rights were explained at the beginning of the online survey. All respondents to the survey were given the option to provide digital informed consent to participate by clicking yes/no before answering the survey questions. All respondents were free to end their participation at any point during the survey.

## 3. Results

### 3.1. Participant Characteristics

In total 550 mothers responded to the questionnaire and were included in the analysis. Most respondents (97%) were 20–39 years old (M = 29, SD = 5.4). Almost all respondents (98%) were married, while 513 (93%) were Saudi. Around two-thirds of mothers (69%) reported having a bachelor’s degree and nearly 29% were not working or unemployed. Around half of the respondents (48%) had a monthly family income ranging from SAR 7000 to SAR 15,000. Almost one-third of the respondents were from the Mecca region (30%), while other areas were represented by fewer than 15 respondents. A total of 133 respondents (24%) had only one child, most (46%) had two children, and 30% had three or more children (Table 1).

Two-thirds of respondents (67%) had babies aged 6 months or younger (M = 5, SD = 3.9). Most (93%) were not pregnant at the time of the survey. A high percentage (75%) had vaginal births, while about 45% reported having difficulties during their last birth (regardless of birth mode). About half (52%) were consistently breastfeeding, while the rest varied from those who used to breastfeed but stopped to those who were inconsistently breastfeeding and those who had never breastfed (12%, 23% and 13%, respectively). More than half (54%) were receiving constant family support, while the rest were roughly divided between those whose family support was inconsistent and those who were not receiving family support at all (Table 1).

A high percentage of respondents (80%) were either within the range of normal or overweight BMI; the rest were considered obese. Around 40% were not performing any form of physical activity, although more than half (56%) had a suitable space at home to do so. Only 13% were going to the gym regularly, while around 60% were not going at all. Smoking was reported by 6.5% of respondents, and most (91%) had not used antibiotics while pregnant. About 11.5% were struggling with at least one chronic health issue. The most frequent health issues reported were diabetes, gastrointestinal, and mental health issues (Table 1).

### 3.2. EDPS Scores

Of the 550 respondents, almost three-quarters (75%) scored 12 or higher on the EPDS, indicating a risk of PPD. The average EPDS score was 14, which was above the cut-off point of 12.

### 3.3. Sociodemographic Factors and Risk of PPD

Family income and area of residence were significantly correlated with the risk of PPD (*p* value < 0.001 and 0.002, respectively). This significance was reflected in the proportion of women with high EPDS scores across income levels. For income levels less than SAR 10,000, the proportion ranged between 83–88%, for higher income levels, the proportion ranged from 48% to 77%. In Dammam, Buraidah, Medina, and Abha, the proportion of women at high risk of PPD was over 80%, while in Riyadh and Mecca, it was closer to 60% (Table 2).

### 3.4. Health Status, Lifestyle Factors, and Risk of PPD

There was a significant association between baby age and risk of PPD (*p* < 0.001). Respondents with babies under 6 months of age had a higher risk of PPD (80%) compared to those who gave birth over 7 months ago (61%). There was also a significant association between birth difficulty and a higher risk of PPD (*p* < 0.001). This significance was reflected in the percentage of respondents with higher scores on the EPDS across the five levels of difficulty of easy/very easy birth experience (over 50%), moderate/difficult experience (over 70%), and very difficult experience (83%). Not receiving family support was significantly associated with a higher risk of PPD (*p* < 0.001). Those who received inconsistent family support were at a higher risk of PPD than those who had consistent or no support (87% compared with 69–73%, respectively) (Table 2).

Those who were more physically active had a higher risk of PPD than those who were inconsistent or not physically active (87% compared to 68–70%, respectively, and this was statistically significant, (*p* < 0.001). We found similar results for going to the gym: a high percentage of respondents who either went to the gym regularly or sometimes had a higher risk for PPD. However, we found that those who had a suitable space at home in which to perform physical activity had significantly less risk (*p* < 0.05) of PPD compared to those who did not (PPD risk was 70% and 79%, respectively) (Table 2).

None of the chronic health issues (mental health issues, diabetes, high blood pressure, heart disease, respiratory allergies, osteoporosis, blood diseases, food allergies, gastrointestinal issues and others including Hashimoto’s disease, thyroid inactivity, stress/tension, rheumatism, sinus sensitivity, lactose sensitivity, or chronic muscle pains) were significantly associated with a higher risk of PPD (*p* > 0.05). Despite a lack of statistical significance, we noted that the proportion of women at risk of PPD among those suffering from health issues was higher than the proportion who were not. However, there was a significant association between being at risk of PPD and suffering from at least one of the diseases put under ‘other’ (*p* < 0.05). People suffering from these diseases had a higher risk of PPD than others (33% compared to 7.5%, respectively) (Table 2).

### 3.5. Predictors of the Risk of PPD Development

In the simple binary logistic regression (univariate analysis), several variables were significantly associated with the risk of PPD (*p* < 0.05). The number of children and birth difficulty were not statistically significant (*p* > 0.05 for the models or the B-coefficient of the independent variables). These two variables were therefore not entered into the further multivariate analysis (Table 3).

In the multivariate binary logistic regression, we found lower income, receiving some support, inconsistent physical activity, and going to the gym (sometimes) significantly influenced the risk of PPD (*p* < 0.05). These factors returned a positive effect except for Physical Activity (Sometimes). The odds > 1 indicate that the chance of developing PPD was greater than not (e.g., in the case of lower income, the chance of PPD was three times greater than not). For Physical Activity (Sometimes), the odds were 0.417 (less than 1), which means that there was a 41.7% risk for PPD (Table 3).

## 4. Discussion

We evaluated various health status, lifestyle, and sociodemographic factors in women and their potential relationship with the risk of PPD within 1 year postpartum. We found that a lower income, difficult birth experiences, lack of consistent family support, and levels of physical activity were significantly correlated with the risk of PPD. Our findings echo other Saudi studies, as 74% of our study sample scored over 14 on the EPDS [13,17]. This could be due to the circumstances of the COVID-19 pandemic, as our survey was conducted between April and November 2021 (during the height of the pandemic) and reflects the critical findings of Papworth et al. (2021) who found that 90% of mothers who had recently given birth felt isolated due to the COVID-19 restrictions, as well as several other international studies that showed that women during COVID-19 were more likely to present clinically significant levels of depressive and anxiety symptoms [7,9,15,33,34].

We found significant associations between the risks of PPD and age, level of education, employment, level of income, and area of residence. This is similar to the findings of previous studies and could be related to social and family dynamics, or the pressures of studying or working while pregnant or mothering [12,13,19,20], as well as the increase in these pressures during the pandemic [15]. This also echoes the findings of other international studies on the effects of the pandemic on maternal mental health [7,9,33]. Our regional findings were interesting, with PPD rates ranging from 57.1% to 89.5%, indicating that at least half of the mothers and a majority of the respondents from these cities may have been struggling with symptoms of depression at the time of the study [13,19,20].

There was a clear significance in the correlation between lower income and the risk of PPD in our study. Lower income could be a factor impacting the availability and accessibility of family support during the postpartum period, as well as access to needed resources to facilitate mothers’ breastfeeding, recovery, and sustained well-being, including engagement in physical activity. Our findings also complement the current literature, as we found high risks of PPD when women were not receiving consistent family support or unemployed [7,9,11,12,13,14,19,43]. We reason that the difference in depression rates among the three levels of family support is as follows: many of those receiving no family support may have become accustomed to this situation. However, many of those who were receiving consistent family support may have also been facing other forms of stress, such as over-involvement of the family or difficulty in communicating or setting boundaries, especially during the pandemic.

Similar to previous studies, we found interesting variations in the risks of PPD in younger age mothers, first-time mothers, mothers with two children, and mothers with more than three children [11,12]. These findings could be related to the lack of experience and awareness of the pressures of motherhood in new and first-time mothers, a lack of social support and the increased amount of care, work and mothering required as the number of children increases [12,16,28] and could be explored further. We also found a significant association between baby age and the risk of PPD, as those with babies under 6 months of age had a higher risk of depression (80% vs. 61%) compared to women whose babies were older. These issues bear further scrutiny and exploration as to the type and amount of support required for a healthy and well postpartum period, which would reduce the risk of PPD.

Our findings for 45% of the study sample who reported difficulties with their last birth are concerning and could be attributed to the COVID-19 restrictions in place at the time, as well as systematic and cultural issues in maternity and obstetrics care [7,9,11,12,14,15,33]. We calculated that the odds of developing PPD after a difficult birth were 11 times higher, which was significant, with a probability of around 91.7%. We also reaffirm the findings linking postpartum medical complications (e.g., postpartum hemorrhage or infections) and difficulties in breastfeeding or childcare, which lead to stress, anxiety, poor sleep quality, fatigue, and increased incidence of PPD [12,14,17,18].

Our results linking physical activity and PPD were surprising and conflict with the currently accepted knowledge [22,23,27]. We found the highest levels of PPD in women who consistently engaged in some form of physical activity. A study from 2020 found little evidence for an association between physical activity and depressive symptoms and discussed the evidence of associations between diet, physical activity, and depression; however, they recommended further research on the relationship between physical activity, specific dietary components, and PPD [24]. In addition, we cannot overlook that the data collection for this study occurred during the COVID-19 pandemic, when severe restrictions were in effect, which may have impacted women’s ability to engage in physical activity or go to the gym and may have confounded mental health issues.

Our study had several strengths as well as some limitations. Although this was a multi-region population-based study with a large sample size, our data were based on a cross-sectional design, and the non-probability convenience sampling method may have hindered the generalizability of the results to all mothers. In addition, our data were collected using electronic platforms and a self-reported questionnaire, which may also have caused selection bias and limitations in the generalizability of our findings. Future studies may consider other methods, such as prospective designs, in-person interviews, or other objective measures to circumvent such limitations.

This study investigated the role of several key maternal health status and lifestyle factors in the risk of developing PPD, yet the data were collected during the height of the COVID-19 pandemic. We did not have information related to the impact of the pandemic on the risk of PPD development among our study sample, as the study design was cross-sectional, and we did not have baseline data for pre-pandemic levels of PPD risk. The pandemic may have played a major role in the risk of PPD found in this study. It was beyond the scope of this study to focus on and directly investigate the pre-pandemic levels of physical activity engaged in by our sample.

### Study Implications and Recommendations

These issues require further research to link women’s integrative and preventive health policies and programs in order to improve reproductive health and maternal mental health [1,2,3,6,8,9,11,12,16,28]. Attention from primary care providers and other healthcare professionals and policymakers working in the reproductive and maternal health fields is also required [21,34,35].

We strongly encourage increased national attention to the issue of maternal mental health and further research exploring the burden of perinatal mental health issues, such as PPD on the well-being of the mother, the baby, family unit, society, and economy. It may also be of value to consider studying the effects of lifestyle factors, such as diet, nutrition, physical activity, and family support, in the context of Saudi Arabia. In light of our intriguing findings on physical activity, further research on participation in physical activity, exercise, and the relationship with PPD risk among new mothers should also be conducted, as exercise has been previously shown to be a cost-effective treatment for improving mental health and well-being and mitigating PPD symptoms [22,23,26]. The impact of a difficult birth experience itself (birth trauma) and the potential emotional impact of the intention to breastfeed, breastfeeding success on perinatal mental health issues, and maternal wellness in general also need to be studied.

## 5. Conclusions

PPD risks were significantly associated with lower income levels, birth difficulty, younger baby age, lack of family support surrounding the mother, and levels of physical activity. PPD is a serious public health concern that has far-reaching consequences for the health and quality of life of the mother and child, as well as for the well-being of the family unit and society at large. Furthermore, it has serious economic implications that must be considered by policymakers. Urgent attention and resources should be directed towards screening and treating PPD in the healthcare system. The development of programs for the education and support of postpartum mothers in the Kingdom of Saudi Arabia is also required.

## Figures and Tables

**Table 1:** Respondent Characteristics (N = 550).

Variable	Categories	Frequency (N)	Percentage (%)
Age	18–19	5	0.9
	20–29	291	52.9
	30–39	242	44.0
	40–50	12	2.2
Marital Status	Married	540	98.2
	Divorced	9	1.6
	Widowed	1	0.2
Nationality	Saudi	513	93.3
	Non-Saudi	37	6.7
Education	No Formal Education	14	2.5
	Secondary or Below	47	8.5
	Diploma	35	6.4
	Bachelor	381	69.3
	Higher Education	73	13.3
Employment Status	Not Working/Unemployed	158	28.7
	Student	32	5.8
	Employed (Public Sector)	150	27.3
	Employed (Private Sector)	179	32.5
	Self-Employed	31	5.6
Family Income (SAR/Month)	<3000	26	4.7
	3000–5000	59	10.7
	5001–7000	134	24.4
	7001–10,000	132	24.0
	10,001–15,000	83	15.1
	15,001–20,000	46	8.4
	20,001–25,000	28	5.1
	25,001–30,000	13	2.4
	>30,000	29	5.3
Area of Residence	Mecca	166	30.2
	Buraidah	75	13.6
	Riyadh	62	11.3
	Al Dammam	59	10.7
	Medina	44	8.0
	Abha	40	7.3
	Other	104	18.9
Number of Children	1	133	24.2
	2	253	46.0
	3	108	19.6
	>3	56	10.2
Baby Age (months)	≤6	370	67.3
	>6	180	32.7
Currently Pregnant	Yes	39	7.1
	No	511	92.9
Birth Mode	Vaginal	413	75.1
	Caesarean	137	24.9
Last Birth Difficulty	Very Easy	23	4.2
	Easy	52	9.5
	Moderate	228	41.5
	Difficult	149	27.1
	Very difficult	98	17.8
Breastfeeding	Yes	286	52.0
	Yes, but stopped	66	12.0
	Sometimes	127	23.1
	No	71	12.9
Family Support	Yes	297	54.0
	Sometimes	125	22.7
	No	128	23.3
BMI	Underweight	16	2.9
	Normal	211	38.4
	Overweight	234	42.5
	Obese	89	16.2
Physical Activity	Yes	154	28.0
	Sometimes	176	32.0
	No	220	40.0
Going to the Gym	Yes	73	13.3
	Sometimes	150	27.3
	No	327	59.5
Adequate Space for Physical Activity at Home	Yes	310	56.4
	No	240	43.6
Smoking	Yes	36	6.5
	No	514	93.5
Antibiotics During Pregnancy	Yes	50	9.1
	No	500	90.9
Chronic Health Issue	Yes *	63 *	11.5
	No	487	88.5

* 15 (24%) had mental health issues, 15 (24%) diabetes, 9 (14%) high blood pressure, 11 (17%) sensitivity, 1 (2%) osteoporosis, 9 (14%) food sensitivity, 13 (21%) gastrointestinal and 9 (14%) had other health issues.

**Table 2:** Chi-square analysis: health status and lifestyle issues vs. PPD risk.

Variable	Category	Frequency (N)	PPD Risk (%)	*p* Value
Mother Age	18–19	5	40.0	0.262
	20–29	291	71.8	
	30–39	242	77.3	
	40–49	11	72.7	
	50–59	1	100.0	
Education	No Formal Education	14	92.9	0.132
	Secondary or Below	47	74.5	
	Diploma	35	71.4	
	Bachelor	381	75.3	
	Higher Education	73	64.4	
Employment Status	Not Working/Unemployed	158	67.1	0.150
	Student	32	81.3	
	Employed (Public Sector)	150	74.0	
	Employed (Private Sector)	179	77.7	
	Self-Employed	31	80.6	
Monthly Family Income (SAR/Month)	<3000	26	88.5	<0.001 *
	3000–5000	59	86.4	
	5001–7000	134	85.1	
	7001–10,000	132	82.6	
	10,001–15,000	83	55.4	
	15,001–20,000	46	47.8	
	20,001–25,000	28	60.7	
	25,001–30,000	13	76.9	
	>30,000	29	51.7	
Area of Residence	Al Dammam	59	83.1	0.002 *
	Al Baha	12	75.0	
	Skaka	15	66.7	
	Arar	23	73.9	
	Riyadh	62	67.7	
	Buraidah	75	86.7	
	Medina	44	79.5	
	Tabuk	17	88.2	
	Jazan	11	63.6	
	Hail	19	89.5	
	Abha	40	82.5	
	Mecca	166	62.7	
	Najran	7	57.1	
Marital Status	Married	540	74.1	0.656
	Divorced	9	66.7	
	Widowed	1	100.0	
Number of Children	1	133	70.7	0.053
	2	253	78.7	
	3	108	65.7	
	>3	56	76.8	
Currently Pregnant	Yes	39	74.4	1.000
	No	511	74.0	
Baby Age	≤6 months	370	80.3	<0.001 *
	>6 months	180	61.1	
Birth Mode	Vaginal	413	75.5	0.177
	Caesarean	137	69.3	
Birth Difficulty	Very easy	23	56.5	<0.001 *
	Easy	52	53.8	
	Moderate	228	77.2	
	Difficult	149	73.2	
	Very difficult	98	82.7	
Breastfeeding	Yes	286	73.8	0.078
	Yes, but Stopped	66	74.2	
	Sometimes	127	80.3	
	No	71	63.4	
Family Support	Yes	297	69.0	<0.001 *
	Sometimes	125	87.2	
	No	128	72.7	
Smoking	Yes	36	72.2	0.844
	No	514	74.1	
BMI	Underweight	16	81.3	0.155
	Normal	211	75.4	
	Overweight	234	69.7	
	Obese	89	80.9	
Physical Activity	Yes	154	87.0	<0.001 *
	Sometimes	176	67.6	
	No	220	70.0	
Going to the Gym	Yes	73	84.9	<0.001 *
	Sometimes	150	84.7	
	No	327	66.7	
Space for Physical Activity at Home	Yes	310	70.0	0.018 *
	No	240	79.2	

Data are presented as frequency (N) and percentage (%). * Significance level at *p* ≤ 0.05.

**Table 3:** Logistic regression: study variables and EPDS scores.

Dependent Variable	Predictors	B	SE	*p* Value	Odds	95% CI (Lower–Higher)
Simple Logistic Regression						
PPD Risk	Lower Income	1.493	0.205	0.000 *	4.450	2.975–6.656
	Constant	0.212	0.143	0.137	1.236	
	1 child (reference)			0.056		
	2 children	0.425	0.245	0.083	1.529	0.947–2.469
	3 children	−0.228	0.278	0.413	0.796	0.462–1.373
	>3 children	0.317	0.369	0.392	1.372	0.665–2.831
	Constant	0.880	0.190	0.000	2.410	
	Baby Age	−0.111	0.025	0.000 *	0.895	0.852–0.939
	Constant	1.643	0.173	0.000 *	5.172	
	Difficult Birth	0.278	0.198	0.159	1.321	0.897–1.946
	Constant	0.926	0.127	0.000	2.523	
	Consistent Support (reference)			0.001 *		
	No support	0.176	0.235	0.453	1.192	0.753–1.889
	Inconsistent support	1.118	0.296	0.000 *	3.057	1.713–5.458
	Constant	0.801	0.125	0.000	2.228	
	Regular Physical Activity (reference)			0.000 *		
	No Physical Activity	−1.055	0.281	0.000 *	0.348	0.201–0.604
	Physical Activity (Sometimes)	−1.166	0.289	0.000 *	0.312	0.177–0.549
	Constant	1.902	0.240	0.000	6.700	
	Not Going to Gym (reference)			0.000 *		
	Going to Gym sometimes	1.016	0.255	0.000 *	2.761	1.674–4.553
	Regular Gym	1.036	0.348	0.003 *	2.818	1.426–5.569
	Constant	0.693	0.117	0.000	2.000	
	Space for Physical Activity at Home	−0.488	0.202	0.016	0.614	0.414–0.911
	Constant	1.335	0.159	0.000	3.800	
	Other Chronic Health Issue **	−1.775	0.714	0.013	0.170	0.042–0.687
	Constant	1.081	0.099	0.000	2.949	
Multivariate Logistic Regression						
PPD Risk	Lower Income	1.121	0.214	0.000 *	3.069	2.019–4.664
	Younger Baby Age	0.525	0.209	0.012	1.691	1.124–2.545
	Consistent Support (Reference)			0.003		
	No Support	0.335	0.259	0.196	1.398	0.841–2.323
	Inconsistent support	1.045	0.316	0.001 *	2.844	1.532–5.279
	Regular Physical Activity (Reference)			0.003		
	No Physical Activity	−0.128	0.230	0.577	0.879	0.56–1.381
	Physical Activity (Sometimes)	−0.874	0.268	0.001 *	0.417	0.247–0.705
	Not Going to Gym (reference)			0.009		
	Going to Gym (Sometimes)	0.851	0.299	0.004 *	2.341	1.302–4.211
	Regular Gym	0.614	0.384	0.110	1.848	0.87–3.922
	Space for Physical Activity at Home	−0.119	0.205	0.561	0.888	0.594–1.327
	Other Chronic Health Issues **	−1.146	0.754	0.128	0.318	0.072–1.393

B: Unstandardized beta coefficient; SE: standard of error. * Significance level at *p* ≤ 0.05. ** Hashimoto’s, thyroid inactivity, stress/tension, rheumatism, sinus sensitivity, lactose sensitivity, or chronic muscle pain.

## Data Availability

The raw datasets generated and/or analyzed during the current study are not publicly available as other related research projects are still ongoing; however, they will be available from the corresponding author on reasonable request.
